# Rationale and design of the DP-TRANSFERS project: diabetes prevention-transferring findings from European research to society in Catalonia

**DOI:** 10.1186/s12967-016-0867-z

**Published:** 2016-04-27

**Authors:** Bernardo Costa, Conxa Castell, Xavier Cos, Claustre Solé, Santiago Mestre, Marta Canela, Antoni Boquet, Joan-Josep Cabré, Francisco Barrio, Gemma Flores-Mateo, Daniel Ferrer-Vidal, Jaana Lindström, The Catalan Diabetes Prevention Research Group

**Affiliations:** Jordi Gol Primary Care Research Institute, Reus-Tarragona Diabetes Research Group, Catalan Health Institute, Primary Health Care Division, Camí de Riudoms 53-55, 43202 Reus-Barcelona, Spain; Public Health Division, Departament of Health, Generalitat de Catalunya, Barcelona, Spain; Chronic Disease Prevention Unit, National Institute for Health and Welfare, Helsinki, Finland

**Keywords:** Diabetes prevention, Prediabetes, Impaired fasting glucose, Impaired glucose tolerance, Study protocol, Public health, Translational research

## Abstract

**Background:**

Compelling evidence has been accumulated to support the effectiveness of intensive lifestyle intervention in delaying progression to Type 2 diabetes even in people identified as being at high risk determined by the Finnish diabetes risk score. The DE-PLAN-CAT project (diabetes in Europe-prevention using lifestyle, physical activity and nutritional intervention–Catalonia) evidenced that intensive lifestyle intervention was feasible and cost-effective on a short scale in real-life primary care settings, at least over 4 years. However, transferring such lifestyle interventions to society remains the major challenge of research in the field of diabetes prevention.

**Methods/design:**

The derived DP-TRANSFERS (diabetes prevention-transferring findings from European research to society) is a large scale national programme aimed at translating a tailored lifestyle intervention to the maximum of primary care centres where feasible through a core proposal agreed with all the partners. The method is built upon a 3-step (screening, intervention and follow-up) real-life, community-wide structure on the basis of a dual intensity lifestyle intervention (basic and continuity modules) and supported by a 4-channel transfer strategy (institutional relationships, facilitators’ workshops, collaborative groupware and programme WEB page). Participation will initially cover nine health departments (7 million inhabitants) through nine coordinating centres located in metropolitan (3.2 million), semi-urban (2.9 million) and rural (0.9 million) areas from which it is expected accessing 25 % of all primary care settings, equivalent to 90 associated centres (1.6–1.8 million people) with an estimate of 0.32 million participants aged 45–75 years at high risk of future development of diabetes. To ascertain sustainability, effect, satisfaction and quality of the translation programme statistical analyses will be performed from both the entire population (facilitators and participants) and a stratified representative sample obtained by collecting data from at least 920 participants.

**Discussion:**

The DP-TRANSFERS will use a strategy of approach to society consistent with the impact of the disease and the fast accessibility provided by primary care settings in Catalonia. Both the widespread effect of the lifestyle intervention and the translational process itself could be assessed.

## Background

Type 2 diabetes is a serious public health problem in continuous increasing during the past decades. The risk factors for the disease are closely related to lifestyle, which is partly an effect of personal choices but also a result of built environment, physical and social background, culture, and socio-economic issues. The most relevant risk factors are obesity, sedentary lifestyle, and unhealthy diet. Therefore, the main drivers of the current epidemic level are increasing obesity—leading to more people developing diabetes during their life-course—and population aging, with more people living longer with impaired glucose metabolism [1].

The notion that diabetes development can be prevented or delayed by intensive lifestyle intervention is not new [2–4]. Since 2007 the International Diabetes Federation recommends a stepwise approach starting with identification of those who may be at higher risk, following with the measurement of current risk and finally, delivering an appropriate intervention [5]. In terms of public health, these theoretical statements would only be sustainable if it really could be first tailored to the specific local situation and then transferred to clinical practice, particularly in primary health care [6]. In fact, it has recently been suggested that progression to diabetes can be also delayed by intensive intervention when applied to real-life primary health care of high-risk subjects identified first with the simple Finnish diabetes risk score (FINDRISC) tool [7].

Despite some controversy [8], it is commonly accepted that a well designed and implemented programme on diabetes prevention is more effective (and even more cost-effective) than doing nothing. Undoubtedly, the efficiency may vary depending on the wealth of the participants but also on their dietary habits and the ability of an intervention to significantly reduce weight [9]. Recent results from Finland have shown that diabetes can be postponed in average by 5 years in people who already have impaired glucose regulation [10]. Even if we could not prevent the development of diabetes, just delaying the disease to later life might have a large impact both on individual and on societal level.

Translational actions are those applying the knowledge from research to clinical practice. Regarding diabetes prevention, any strategy intended at national level should be feasible, effective and efficient, issues that should be evidenced in advance. This was the original background of the international European DE-PLAN project (diabetes in Europe-prevention using lifestyle, physical activity and nutritional intervention) [11] in which the Catalan research associated group (DE-PLAN-CAT) reached to ascertain long-term feasibility, effectiveness and cost-effectiveness of the lifestyle intervention at national level [12, 13].

The project was conducted in 18 primary care centres with an optimum positive response rate (greater than 82 %) for both screening (n = 2054) and intervention (n = 552). The overall incidence of diabetes was reduced by 36.5 % at 4-year follow-up in participants receiving the intensive intervention compared to the standard care regime [12]. Additionally, a convenient cost-effectiveness ratio (3243 € per QALY gained) could be clearly shown [13]. Similarly to most well designed clinical and implementation programmes, the DE-PLAN-CAT used traditional lifestyle intervention modes such as individual and group counselling [14, 15]. Indeed, this model may be really feasible and cost-effective on a short scale in primary care, but if the number of participants (intervention exposure) is low, a high effect on the community as a whole should not be expected. Following the steps provided for this national programme, a set of translational actions were planned in order to involve those Catalan primary care centres who agree to participate.

The rising DP-TRANSFERS (diabetes prevention-transferring findings from European research to society) project was defined and structured as a translational programme aimed at transferring the DE-PLAN-CAT knowledge, methodology, didactic materials and results—if feasible—to daily clinical practice in primary health care. Both the widespread effect of such lifestyle intervention and the translational process itself could be assessed.

## Design and rules

### Hypothesis

Transferring a cost-effective strategy in delaying progression to Type 2 diabetes among high-risk Mediterranean individuals (DE-PLAN-CAT) is also feasible on a large scale in primary care using existing public healthcare resources.

### Objectives

#### Primary objective

The primary objective is to asses the feasibility and effect of the DE-PLAN-CAT intensive lifestyle intervention when translating into society through a central process of dissemination agreed with the primary care centres.

#### Secondary operational objectives

(a) To establish, expand and reinforce a multidisciplinary Steering Committee with representatives from primary care coordinating centres (at least one of each health department involved) to implement a single common translational protocol as well as a curriculum for the training of prevention managers (nurses and general practitioners).

(b) To identify needs, design and adapt the DE-PLAN-CAT intensive lifestyle intervention to the structural conditions in primary care settings determinants of the real-life clinical practice interventions which are associated with and could predict a beneficial outcome.

(c) To develop a specific set of easily accessible didactic material for such lifestyle intervention (presentations, information sheets and feed-back exercises) in conventional and digital format, from own files but also from European projects on diabetes prevention in which the Catalan group is associated.

(d) To assess the sustainability and quality of translation process through the evaluation of the resources (balance and cost), the actions (intervention effect) and the opinion of the target population (facilitators and participants).

### Study design

The project does not have a conventional design since it is a sequential and coordinated set of actions to be performed in primary care in order to achieve a reasonably effective translation of the DE-PLAN-CAT lifestyle intervention using the available resources efficiently.

### Setting

The original DE-PLAN-CAT project was performed in 18 primary health-care centres (Catalan Health Institute). The derived DP-TRANSFERS programme will translate a tailored lifestyle intervention to the maximum of primary care centres where feasible.

### Procedure and sample size calculation

**Fig. 1:**
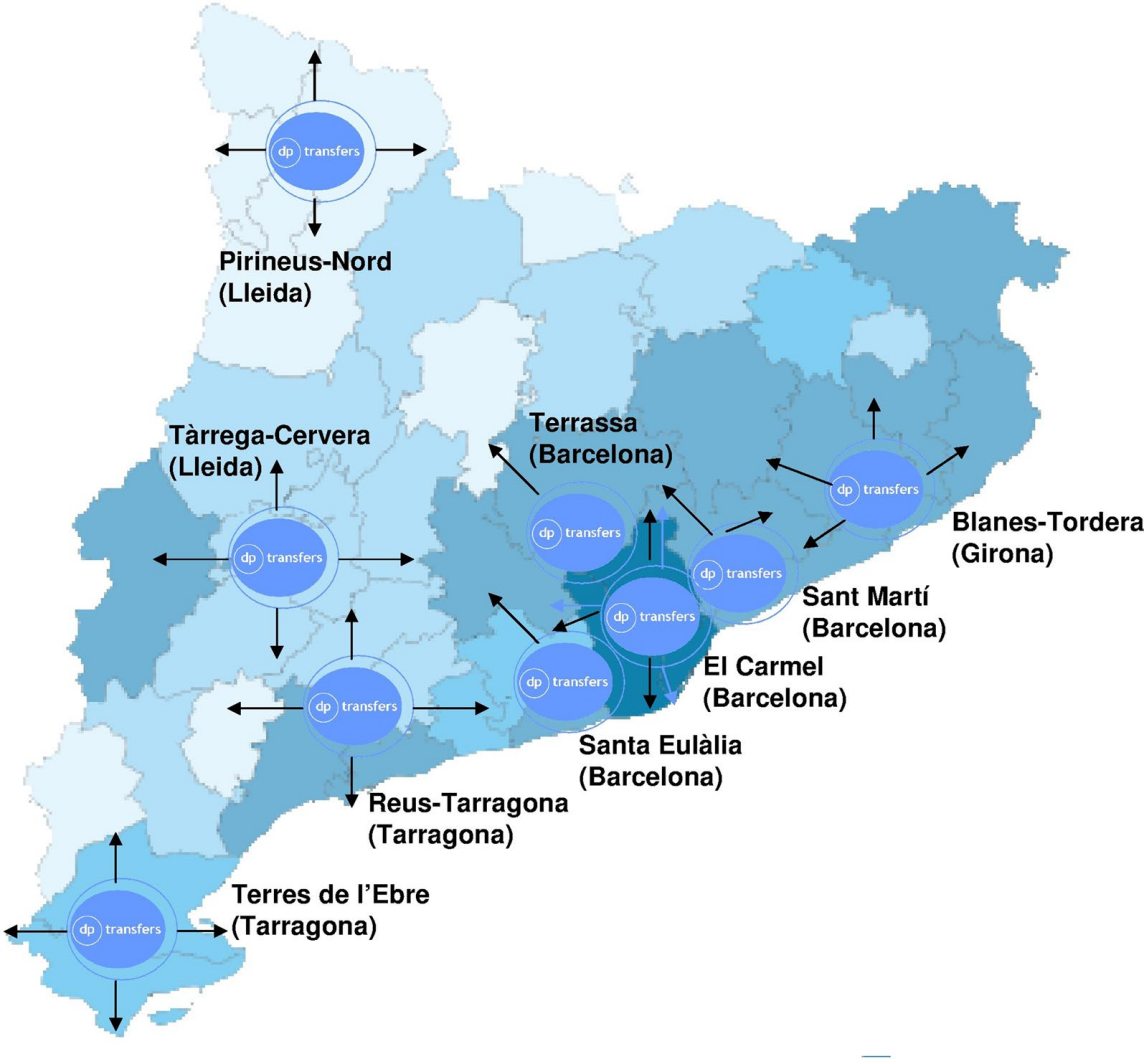
Geografical distribution of the DP-TRANSFERS coordinating centres at the start of the programme; The *colour* intensity is proportional to the absolute density of population per county within the Catalan territory

The sample size calculation details concerning feasibility and cost-effectiveness of the DE-PLAN-CAT have been published previously [12, 13]. Participation in the DP-TRANSFERS will initially cover nine public health departments (reference population: 7 million inhabitants) through nine coordinating centres located in metropolitan (3.2 million), semi-urban (2.9 million) and rural (0.9 million) areas (http://www.gencat.cat/ics/usuaris/centres_serveis.htm). The programme aims to involve the largest number of participants (subjects at diabetes risk) as possible. However, only data from a representative sample of participants will be used for evaluating the translation process and the results. The proposal for participation will be first agreed with the managers and staff of primary care centres. Thus, considering a tree structure starting from each coordinating centre, the new participating ones (associated centres) will be invited in a stratified manner following a representative geographic distribution as well as particular responsibilities within the health system (Fig. 1).

The maximum level of participation is determined by a total of 369 pre-existing centres, 334 (90 %) ascribed to the Catalan Health Institute who attended 4.2 million people during the last year 2014 [16]. Assuming that involving all these centres simultaneously would be ineffective, the Steering Committee approved expanding lifestyle intervention to a 25 % of the primary care centres in order to have a significant impact on the whole community in 3 years (1 year for the screening step and 2 years for the intervention step). Regarding a progressive shifting process, it is expected to involve at least 5 % of new centres during the first year of programme implementation and not less than 20 % over the next 2 years which would mean a total of 90 primary care centres (reference population: 1.6–1.8 million inhabitants and, hypothetically, 0.32 million classified as having diabetes risk by the FINDRISC).

Although the effectiveness of the lifestyle intervention is already proven, an effort will be made to ascertain sustainability and quality of the translation process. For this purpose, statistical analyses will be performed from a stratified sample. As for the risk screening step, data entry will be restricted to the 5, 10 and 15 first consecutive participants with positive screening invited to receive the intervention, on the basis of three strata: rural (<5000 inhabitants), semi-urban (5000–10,0000) and metropolitan (>100,000). Similarly for the intervention step, baseline and 2-year follow-up data entry will be restricted to the 5, 10 and 15 first consecutive participants who ultimately accept to receive the lifestyle intervention.

Thus, accepting a proportional sample distribution of the attended population within these strata and allowing a two-year discontinuation rate close to 20 % (similar to that found in the DE-PLAN-CAT study), it is expected collecting data from at least 920 participants in the screening (118 rural, 381 semi-urban, 421 metropolitan) and 736 participants in the subsequent lifestyle intervention to test feasible and effective ways for assessing diabetes risk, delivering the intervention and providing support to maintain successful behavioural changes (statistical power 82.5 %; type 1 and type 2 errors 5 and 20 %, respectively).

## Methods and participants

### Facilitator level (health professionals)

**Fig. 2:**
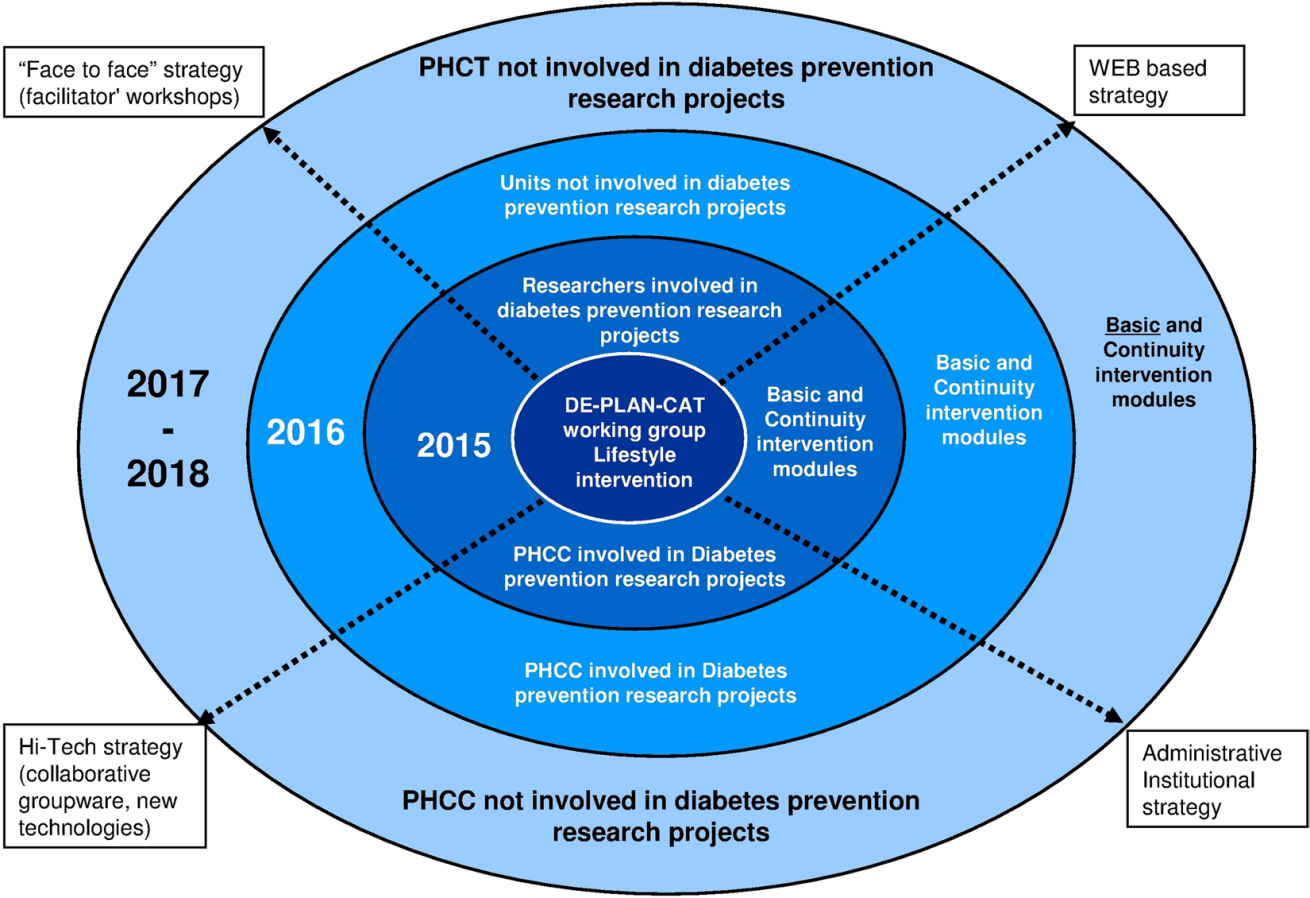
DP-TRANSFERS chronogram and strategy steps. *PHCT* primary health care team, *PHCC* Primary Health Care Centre, DE-PLAN-CAT (diabetes prevention program project)

The working method of the DE-PLAN-CAT public health study will be used for the translational programme purposes [12]. Additionally, the multidisciplinary Steering Committee has been reinforced to implement the DP-TRANSFERS initiative with representatives from each coordinating centre (nurses and general practitioners), epidemiologists, dieticians and experts in facilitating health interventions within a community setting. This coordinating group will be also shared by executive staff of the leading providers of public health services, technicians of the government agency (Department of Health) and also by relevant members from primary care scientific societies. The current priority is to translate the DE-PLAN-CAT compelling experience into clinical solutions by supporting first a 3-year, 3-stage transfer procedure (Fig. 2). The three stages designed include: (a) Involvement of primary care teams already working on diabetes prevention; (b) Involvement of the rest of primary care teams who have not previously participated in tasks specifically related to diabetes prevention in those centres; (c) Involvement of new teams operating in other primary care centres with the common characteristic of never having participated in regulated projects aimed at preventing diabetes.

The translation process will be conducted through four channels or strategies (Fig. 2): (a) Specific website which will also include an electronic case report form (eCRF) for data collection; (b) Face-to-face workshops for facilitators prior to the start of any intervention. Members of the Steering Committee will widely inform on the programme, delivery rules, training tools and evaluation criteria; (c) Administrative structures of each institution will be linked and implemented; (d) High technologies, videoconferencing and collaborative groupware will be used for information exchange. A persuasive internet-based interactive system to facilitate self-monitored intervention is also planned but would be part of a second phase as well as translation towards built environment (municipalities, workplaces, pharmacies and non-governmental organizations) trying to promote screening actions, physical activity and healthy diet.

The method of transferring the intervention will include assistance in increasing participant’s motivation to change lifestyle, planning actions and promoting persistence over time. Changing behaviour is a complex process but it is acknowledged that simply disseminating information about recommended lifestyle is not enough. Recently there has been a shift in diabetes prevention rules, increasing the use of techniques that facilitate empowerment—the participants no longer being “subjects” of the intervention but equal partners, taking charge of their own way of life. Facilitators may help the process by making questions, suggestions and supporting the change. These will be the key messages introducing nine kick-off training meetings for facilitators to be organized in each coordinating centre.

Design of lifestyle intervention will be conducted by the Steering Committee, particularly nursing. Following the recommendations of the European guideline and training standards for diabetes prevention panel, the intervention has been raised in two levels [17]. First, an individual level for personal goal setting, maintain motivational support and ways to solve relapses. Secondly, a group intervention is required to consolidate the changes in habits and behaviours thereby trying to delay disease progression. The contents of the group sessions will be clearly addressed through an understandable language and positive messages. The method should be as participatory as necessary to involve the attendees from the start of each session.

The core intervention materials (identical for each participating centre) will be provided centrally to all facilitators. The axis of every session consists of a slide set, activity sheets and homework tasks for participants as well as teaching and introductory material for facilitators. Other local equipment may be used as long as they are in line with the DP-TRANSFERS intervention goals. Every group session shall cover relevant aspects of diabetes and cardiovascular risk prevention and especially are thought to encourage a healthy relationship towards eating and exercising. The dietary intervention will focus on the healthy Mediterranean diet pattern promoting consumption of omega-3 and monounsaturated fatty acids [18]. The physical activity intervention will be based on information synthesis and training, if feasible.

### Participant level (subjects at diabetes risk)

Among all users of public primary health care services, the target population for the programme corresponds to people without a diagnosis of Type 2 diabetes between the ages of 45 and 75 years, having either or both of the two following conditions:

1. Diabetes risk suggested by a FINDRISC > 11
2. Prediabetes diagnosis as defined by the World Health Organization diagnostic criteria [19], based on previous (last year) or current (screening time) laboratory reports:
  - Impaired fasting glucose (IFG): fasting plasma glucose (FPG) greater or equal than 6.1 and less than 7.0 mmol/l.
  - Impaired glucose tolerance (IGT): FPG less than 7.0 mmol/l and 2 h- postload glucose (2hPG) in the 2-h 75-g Oral Glucose Tolerance Test (OGTT) greater or equal than 7.8 mmol/l and less than 11.1 mmol/l.
  - IFG plus IGT (both diagnostic categories simultaneously).

All individuals with severe psychiatric disease or serious disorders that could influence the screening or induce discontinuation of the intervention should be excluded at the discretion of facilitators.

The whole programme will be translated and should be integrated into daily clinical practice of the participating primary care centres. The main pre-requisite is the practice having a computerized patient record system. Potential participants can be contacted by letter, telephone or otherwise for a first evaluation. If the centre has already an ongoing strategy for diabetes prevention, computerized clinical records could be reviewed searching for DP-TRANSFERS eligible participants.

The research ethics committee board at the *Jordi Gol* Research Institute (Barcelona) approved the protocol (January 2015), and all participants must give written informed consent.

## Clinical intervention

### Screening step

Opportunistic screening will be the main route of entry into the programme. An advance search from the computerized records of the public health system has been recommended to select potential candidates for screening.

The first screen option will use the Catalan and Spanish versions of the FINDRISC, a well-validated, eight-item European questionnaire related to diabetes risk factors to characterize subjects according to their future risk of Type 2 diabetes. The feasibility and performance of this test have been widely assessed in our country [20, 21]. The most recent version will be the one used, and ranged from 0 to 26 points, as follows: <7 points (low), 7 to 11 (slightly increased), 12 to 14 (moderate), 15 to 20 (high), and over 20 (very high) [10]. The questionnaire collected information about age, sex, weight and height (to calculate body mass index), waist circumference, use of blood-pressure medication, history of high blood glucose disorders, physical activity, family history of diabetes, and daily consumption of vegetables, fruits and berries.

The second screen option refers to the use of previous (last year) or current (screening time) laboratory reports. This option may involve but are not limited to the FPG measurement—the most commonly used in routine clinical practice—since the standardized 2-h 75-g OGTT (along with measurements of FPG and 2hPG) has been also accepted according to the real possibilities of development in the participating centres. For this part of the DP-TRANSFERS programme (screening), diagnosis of all glucose disorders will be based on the results of a single test.

Any potential candidate with either a FPG or 2hPG result suggestive of diabetes will be excluded from participation in the subsequent part of the programme (lifestyle intervention). A second test to confirm the diagnosis of diabetes has been recommended for those individuals who ultimately participate in the intervention and the follow-up.

### Lifestyle intervention step

People will be eligible for the lifestyle intervention only if they did not have diabetes and have either or both of a FINDRISC score >11 or the diagnosis of prediabetes defined by the WHO criteria. All eligible participants with positive screening will be offered the intensive lifestyle intervention which will be usually provided by trained nursing staff. The intervention goals will be similar to those that were recommended by the European DE-PLAN–IMAGE experts’ panel in accordance with the Finnish diabetes prevention study [15, 17]. Thus, the five targets will be: (a) No more than 30 % of daily energy from fat, if feasible; (b) No more than 10 % of energy from saturated fat; (c) At least 3.6 g/1000 kJ (15 g/1000 kcal) of fibre: (d) At least 30 min/day of moderate physical activity and (e) 5 % weight reduction (a more realistic 3 % could also be acceptable).

The 2-year DP-TRANSFERS lifestyle intervention will consist of a synchronous 4 individual counselling sessions and 16 group sessions planned in two different intensities. The core of the group intervention is a 9-h basic module including the first six sessions—to be delivered during 2 months—in groups of 5–15 participants who should also receive specific training materials. A subsequent 15-h continuity module with the remaining ten sessions—to be delivered during 22 months—will complete the scheduled intervention (Table 1).

**Table 1:** Technical development of the DP-TRANSFERS lifestyle intervention

Chronogram (months)	0			2						12						24
FINDRISC assessment	X															
Anthropometric data	X	X								X						X
Laboratory test	X	X								X						X
Informed consent		X														
eCRF data entry		X		X						X						X
Individual intervention		X		X						X						X
Group intervention			S (1–6)		S7	S8	S9	S10	S11		S12	S13	S14	S15	S16	
Sessions’ assistance				X						X						X
MEDAS questionnaire		X								X						X
IPAQ questionnaire		X								X						X
EQ-5D-5L questionnaire		X								X						X
Satisfaction questionnaire				X						X						X
Intervention step/module	Screening		Basic	Continuity (first year) continuity (second year)												

*FINDRISC* Finnish diabetes risk score, *eCRF* electronic case report form, *S* group sessions, *MEDAS* Mediterranean diet adherence screener (from PREDIMED study), *IPAQ* 7-item international physical activity questionnaire, *EQ-5D-5L* 5-item preference-based health-related quality of life instrument

The method for the basic module will be adapted to the experience, needs and skills available based on motivation, peer-support and positive feedback. The following contents have been programmed: (a) What Type 2 diabetes is and what it means to be at risk; (b) Weight management; (c) The Mediterranean diet: nutritional advice based on the “*Prevención con Dieta Mediterránea*”–Mediterranean Diet Adherence Screener (PREDIMED–MEDAS) questionnaire as a tool to increase adherence during the follow-up [22]; (d) Physical activity and its beneficial health effects; (e) Eating control with relapse management and (f) Stress management and tobacco advice (centres that include smokers in the intervention group). The nursing staff taking over the basic module will have a checklist available for the group sessions giving objectives, a brief description of the session including the topics, activity listing, and homework listing.

The method for the continuity module will be focused on maintaining motivation for preventive lifestyle changes by regular group follow-up counselling sessions with an approximate frequency of one session every 2 months (Table 1). In addition, periodic contacts by phone or text message will be allowed. One major objective is to repeat the most important lessons from first six sessions sharing experiences from the basic module and also expanding educational contents as far as possible: (a) Attentive eating; (b) Energy content of food; (c) Learned hunger reactions and acquired habits; (d) Different types of carbohydrates, lipids and proteins and (e) Expanding concepts on physical activity.

The sessions of this module will be associated with individual visits for goal setting and to follow the progress. The facilitator may also use the programme questionnaires as basis of the conversation and goal-making process, if applicable. Process-based evaluation of the individual risk and response must be provided to encourage the lifestyle modification.

## Measuring instruments, outcome measures and evaluation

The evaluation of the programme will focus on three main aspects: (a) Impact on clinical and behaviour indicators commonly associated with a decreasing in diabetes risk; (b) Programme sustainability by assessing process carried out (operational objectives, outputs, outcomes and deliverables) and (c) Economic analysis of direct costs incurred by the implementation of the programme.

For evaluation purposes, a set of measurements will be applied either to the entire participant population or a representative sample through 4 individual visits scheduled at 0, 2, 12 and 24 months in view of detecting participants’ baseline status and possible future changes. During visits, neither feedback nor comments (positive or negative) on the answers will be given. Preferably, the checking should not be done by the facilitator, as the participant may adapt their answers to look more favourable than the true behaviour.

Clinical parameters to be considered and health profile instruments to be used could be summarized as follows (Table 1).

### Impact on clinical and behaviour indicators

#### Evaluation of lifestyle habits

Through three short, well-validated questionnaires: the FINDRISC, the 7-item International Physical Activity Questionnaire (IPAQ) [23] and the 14-item PREDIMED tool (adherence to a Mediterranean diet) [22]. Additional questions on socio-demographic characteristics, concomitant diseases and medications, toxic habits and social support will be asked at the baseline, 12 and 24 month.

#### Non-invasive clinical and anthropometric measurements

Weight, height, waist circumference, blood pressure and heart rate will be measured and recorded using standard methods as a part of routine clinical practice in primary care.

#### Biological measurements

Specifically monitoring glucose-related parameters (FPG, 2h-PG, HbA1c) and lipid profile, particularly serum cholesterol and fractions, would be highly recommended depending on the real possibilities of each participating centre. These measurements will be repeated at the yearly follow-up visits to ascertain Type 2 diabetes incidence. It will be emphasized that programmed biological measurements should not increase the burden of laboratory tests rather they should be part of routine practice in primary care.

#### Psychological and quality of life measurements

Prior to any intervention, the participants must complete a minimal survey focused on individual self-reported interest in introducing lifestyle changes. Quality of life will be assessed using a 5-item preference-based health-related quality of life instrument EQ-5D-5L, a standardised tool for use as a measure of health outcome applicable to a wide range of health conditions that provides a simple descriptive profile and a single index value for health status [24].

In addition, personal satisfaction with the lifestyle intervention and didactic materials will be evaluated using a 7-item specifically-designed questionnaire to be fulfilled both by the facilitators of the intervention and participants.

### Programme sustainability

Analyzing proceedings is an essential element for evaluating the entire DP-TRANSFERS programme. For this purpose there is provided a detailed review of the process in order to assess whether the actions are implemented as planned, achieves operational objectives and whether translation to primary care centres is really feasible. Running objectives and evaluation components (process indicators, outputs, outcomes, impact, timeframe and deliverables) are shown in Table 2.

**Table 2:** Operational objectives and evaluation components for the DP-TRANSFERS translational programme in Catalonia

	Operational objective	Process indicators	Output indicators	Outcome/impact indicators	Timeframe	Deliverables
1	To establish a PHC representative multidisciplinary SC to implement a single common translational protocol and a curriculum for the training of prevention managers	Involving at least 2 professionals (nurse and GP) in each coordinating centre Involving endocrinologist, epidemiologist, dietician and experts in developing health interventions within a community setting	How the SC is working on? Assessment of SC practice staff understanding and commitment with the programme Regularly scheduled SC meetings Focus group interview with SC members	Face-to-face workshops for facilitators designed Administrative structures of each involved institution implemented Collaborative groupware implemented	Ongoing 2015–2018	Annual scientific and financial report Minutes of the SC meetings Working guidelines issued
2	To identify needs, design and adapt the DE-PLAN-CAT intensive lifestyle intervention to the structural baseline conditions in primary care settings	Prioritizing determinants of the real-life clinical practice interventions which are associated with and could predict a beneficial outcome (from DE-PLAN-CAT experience)	Interviews and focus groups with practice facilitators staff developed Predictors of success identified Participants’ barriers to attendance at group sessions identified	Number and type of PHC participating centres Facilitators’ workshops development in-depth analysis Perceived level of collaboration	Ongoing 2015–2016	Intervention manual Minutes of the meetings and workshops
3	To develop a specific set of easily accessible didactic material for the lifestyle intervention (presentations, information sheets and feed-back exercises)	Adapting from own files in conventional and digital format Personnel using didactic material Facilitator and participant opinion Pilot group intervention (testing materials in real practice). Required adjustments	To what extent the material is used by facilitators and participants? Focus group with lifestyle officers Data set about the use of the material ready for analyses Feedback on teaching materials Maintenance in programme	What features were considered useful/not useful by the users? Proportion of facilitators and participants who would recommend the didactic material Proportion of personnel who would continue to use the material	Ongoing 2015–2016 3 months after group sessions were started	Core intervention materials delivered Online set of didactic material available
4	To assess the sustainability and quality of translation process (representative sample) through the evaluation of the resources (balance and cost); the actions (intervention effect) and the opinion of the target population (facilitators and participants)	Specific WEB and eCRF built Facilitators trained for programme Facilitators with opinion evaluated Participants evaluated for eligibility Participants starting the intervention Participants with complete follow-up Participants with opinion evaluated Statistical analyses-participants’ database	Number of PHC teams involved Participants achieving lifestyle changes (at least 2 or more prefixed intervention goals) Background, psychosocial and clinic data collected QoL and opinion collected Inventory of resources used	Changes in dietary patterns and physical activity from baseline Changes in clinical risk factors (body weight, PG, HbA1c, cholesterol) from baseline Economic analysis (based on direct cost of resources used) Satisfaction and QoL analysis	2016-2018 Towards the end of the programme (24-month follow up)	Specific WEB and eCRF ready for continued use. Report on the intervention effect Geographic map of the translation Publishing results

*PHC* primary health care, *SC* Steering Committee, *GP* general practitioner, *DE-PLAN-CAT* diabetes in Europe-prevention using lifestyle, physical activity and nutritional intervention-Catalonia, *eCRF* electronic case report form, *QoL* quality of life, *PG* plasma glucose, *HbA1c* haemoglobin A1c

### Economic analysis

This translation programme is preceded by a confirmatory cost-effectiveness analysis [13]. Thus, the specific DE-PLAN-CAT approach for cost assessment will be also incorporated at least to calculate the average cost per participant in the DP-TRANSFERS programme as well as the economic burden that may represent spreading of the lifestyle intervention to all primary care centres. For the analysis of direct costs generated by the process and the intervention, the primary care centres will be requested to record data on resources used from the representative sample of the participant population whose economic parameters form part of the follow-up and are also included in the eCRF.

Particular attention will be paid to the direct costs incurred during two periods and at two levels. The start-up period cost covers pre-implementation phase, planning, consensus meetings, training, printing and spreading didactic materials. These costs are spent only once for the organization. The post start-up period cost covers implementation and running the programme (screening, intensive intervention and continuous intervention). Cost at management level consider resources used for coordinating of the intervention in each participating centre including planning, organizing and monitoring of the intervention and training of the personnel. Costs at participant level include all resources used at the point of delivery of the intervention such as: costs of screening, blood testing and didactic materials used. Changes in use of health services due to intervention will be also considered.

## Organization, data collection and analysis

### Organization

A multidisciplinary coordinating committee has been established to implement and coordinate the overall translation programme. Two representatives from each coordinating centre (nurse and general practitioner) will be invited to join the coordinating committee. Each of these reference centres would lead a local coordinating commission with representatives from their respective primary care associated centres, drawn out a tree structure. Every providing primary care centre is composed of many independent teams managing their own activity though coordinated at a centre level.

### Data collection

Data will be collected and monitored electronically. Participants will be assigned a unique number (lowest available number allocated to the site) which will remain the same throughout the programme. The Reus-Tarragona coordinating centre will provide, besides coordination and steering, methodological support and statistical data treatment. To assist data collection and subsequent global spread of this initiative, it was developed a website-based eCRF (http://www.faqcil.com/crd/index.php). The main advantages are: centralized data, easy access to the active participants, simple recording, and fast access to programme files, documents and didactic material for lifestyle intervention, fast internal messaging system and a convenient updated consultation at any time. Additionally, it supports different user profiles, enables to backup data allowing confidentiality and multiplatform access.

Data will be directly collected by the associated investigators and revised by an independent non-epidemiologist. To ensure quality and avoid discrepancies, files will be also reviewed by an expert epidemiologist if necessary. In case of data inconsistencies, the teams will be required to complete a “query–response” electronic form. Additional checking will be performed after entering the information to avoid inconsistencies due to any mistake in data input.

### Analysis

Analysis at 12 and 24 months will determine the degree that translational programme objectives were achieved, through overall and sub-group testing. Multiple comparisons of significant differences among groups will be carried out by one-way ANOVA and/or by Student’s t test. Participants who discontinued the programme will be considered to be at risk for diabetes until their last visit, at which point data will be censored. The level of statistical significance will be set as p < 0.05 for all analyses.

Primary outcomes which will be reported include changes in lifestyle habits as physical activity levels and dietary pattern (evaluated through programme questionnaires); clinical and anthropometric measurements (possible reduction in weight, waist circumference and blood pressure); biological measurements (with special emphasis on the potential reduction in diabetes incidence) and finally, personal satisfaction, psychological and quality of life measurements (evaluated through programme preference-based, health-related instruments). Regarding prefixed goals for lifestyle intervention, characteristics and proportions of the cohort achieving 1, 2, 3, 4 or all 5 goals will be analyzed. Logistic regression analysis will be used to determine factors associated with the achievement of programme goals.

Process analyses which will be reported include detailed information on programme reliability and knowledge by staff, satisfaction of participants and facilitators as well as barriers associated with programme implementation and delivery (Table 2). Meanwhile, the economic analysis is not intended to confirm the economic viability of the lifestyle intervention as it has already been demonstrated. The main purpose is to analyse the direct average cost per participant and then estimate the overall cost of the translation process. Direct costs will be accounted for and measured according to reliable available rates in the Catalan health service which provides internal and external consistency. If feasible, a cost-utility analysis could be developed based on utility measures extracted from the EQ-5D-5L questionnaire to obtain the quality-adjusted life-year gained in lifestyle intervention.

## Discussion and points of interest

Transferring sustainable and effective lifestyle intervention to society remains the major challenge of scientific research in the field of diabetes prevention. A recent survey estimated that about 21 % of Spanish adult population could be classified as having undiagnosed prediabetes or Type 2 diabetes [25]. Therefore, a large group of middle-aged individuals could benefit from this proposal. It was recently found that three out of four potential users of public health services have made a consultation to the primary care team at least once a year [16]. Thus, the present translational programme will use a strategy of approach to society consistent with both the impact of the disease and the fast accessibility to health services provided by primary care settings in Catalonia.

The findings from the European DE-PLAN-CAT project should support the conclusion that intervention programmes that have been developed and scientifically tested, not only by academic clinical trials but by primary care implementations must be recognised as the standard evidence-based healthcare. The DP-TRANSFERS proposal aims to utilize our experience from clinical studies, implementation initiatives and development projects, to build up, test, disseminate and promote the uptake of a new nationwide translational programme. Even more, all these transfers’ actions are aimed at efficient prevention of diabetes, in people most at risk, from the perspective of the provider of public health services. Consequently, it would be helpful that consistent preventive measures should be properly planned [26].

Assuming that the programme reaches a truly operational screening loop, the final success mainly depends on ensuring a strong lifestyle intervention [27]. It has been documented that determinants of success are precisely related to a long duration of the first step (basic module) and then a sustained effect of the subsequent reinforcement (continuity module) [28]. In any case, this is one of the first attempts to assess a transfer mechanism to extend a lifestyle intervention to prevent Type 2 diabetes at a national level through a community-wide real-life programme agreed with all primary care centres, an aspect on which there is almost no scientific information available.

### Institutional and financial support

The original DE-PLAN-CAT project was funded by the Commission of the European Communities, Directorate C-Public Health (Grant agreement no. 2004310), by the Institute of Health Carlos III, Spanish Ministry of Health (grant agreements FIS PI05-033 and PS09-001112), and the Department of Health, Generalitat de Catalunya. Current translational DP-TRANSFERS project has been funded by the Institute of Health Carlos III, Spanish Ministry of Health (grant agreements FIS PI14/00122 and PI14/00124) and the European Regional Development Fund (ERDF/FEDER). The kick-off meeting and initial presentation of educational material has the support of Boehringer Ingelheim, Spain.

## Abbreviations

FINDRISC: Finnish diabetes risk score
DE-PLAN: Diabetes in Europe-prevention using lifestyle, physical activity and nutritional intervention
DP-TRANSFERS: diabetes prevention-transferring findings from European research to society
eCRF: electronic case report form
IFG: impaired fasting glucose
IGT: impaired glucose tolerance
FPG: fasting plasma glucose
2hPG: 2 h plasma glucose
OGTT: oral glucose tolerance test
HbA1c: hemoglobin A1c
PREDIMED–MEDAS: *Prevención con Dieta Mediterránea*–Mediterranean Diet Adherence Screener
IPAQ: international physical activity questionnaire
